# Egg donation in the age of vitrification: A study of egg providers’ perceptions and experiences in the UK, Belgium and Spain

**DOI:** 10.1111/1467-9566.13590

**Published:** 2022-11-28

**Authors:** Sara Lafuente‐Funes, Christina Weis, Nicky Hudson, Veerle Provoost

**Affiliations:** ^1^ Goethe University Frankfurt Frankfurt Germany; ^2^ De Montfort University Leicester UK; ^3^ Universiteit Gent Ghent Belgium

**Keywords:** assisted reproduction, bioeconomies, commercialisation, cryopreservation, donor experience, egg donation, IVF, vitrification

## Abstract

IVF treatment involving donated eggs increases yearly. Numerous technical and commercial transformations have reshaped how eggs are retrieved, stored and managed. A key transformation is vitrification; a ‘fast freezing’ method that allows efficient preservation of eggs, and therefore more flexibility in use, giving rise to new commercial possibilities. There has been limited focus on egg providers’ experiences in the context of vitrification and related commercialisation. We report findings from a study in the UK, Spain and Belgium, where we interviewed 75 egg providers. Comparing experiences within different donation ‘regimes’ allows an exploration of how varying national practices and policies shape information‐giving and women’s experiences. In the UK, a system of ‘informed gift‐giving’ was described, where egg providers saw their actions as not‐for‐profit and felt relatively well informed. In Belgium, the system was presented as ‘trusted tissue exchange’: with less information‐giving than in the UK, but clinics were trusted to act responsibly. In Spain, a ‘closed‐door, market‐driven’ system was described, whereby egg providers received little information and expressed concerns about generation of excess profit. Our findings extend understandings of how egg donation is managed at the national level and how donation regimes produce specific experiences, expectations and subjectivities amongst tissue providers.

## INTRODUCTION

Since the birth of the first baby from egg donation in 1984, the use of third‐party eggs has grown, in many parts of the world, from a minority practice to a routinised means of achieving pregnancy. IVF treatment involving the use of donated eggs now accounts for 7% of all treatment globally, and the number of cycles increases annually (Banker et al., 2021). A number of recent technical and commercial transformations have reshaped how eggs are retrieved, stored and managed (Hudson et al., 2020; Waldby, 2019). Alongside these shifts, differences in how egg donation is framed as a social, economic and moral practice have emerged, accompanied by a global patchwork of related policies. In some places, there are few specific regulations governing the use of third‐party eggs (e.g., in Russia and the USA), in others, egg donation is permitted subject to certain conditions (e.g., anonymity in Spain), and in others it is completely outlawed (e.g., in Germany). Wide international variation means that practices related to, for example, the storage of eggs or the number of children that can be born from their use, have evolved differently between jurisdictions. Moreover, despite regulation designed to explicitly curtail the commercialisation of eggs in some contexts—for example, the EU Tissue and Cells Directive in Europe—the growth in egg donation is increasingly shaped by global market forces (Daniels & Heidt‐Forsythe, 2012; Payne, 2015; Waldby, 2019).

A key technical transformation that has affected egg donation is vitrification (Hudson et al., 2020; Waldby, 2019); a freezing method that allows more efficient preservation of the cell. Prior to the development of vitrification, egg donation mostly involved fresh cycles (Argyle et al., 2016), whereby the egg provider and recipient/s needed to have their cycles synchronised in order for IVF to be conducted immediately after the eggs were retrieved. Whilst use of frozen eggs for donation varies by country and clinic, data show that in general the practice of vitrification is growing (Shenfield et al., 2017). This development has allowed clinics increased flexibility with regard to the use and management of eggs (Hudson et al., 2020; Waldby, 2019). One aspect of this flexibility is the ‘batching’ of eggs from one retrieval cycle. This means that either some eggs can be used immediately in a fresh cycle and the remainder vitrified and stored (for the same recipient or additional recipients), or for all eggs from one cycle to be immediately batched and frozen for later use by multiple recipients. This allows increased flexibility in storage and use, for example, by the creation of in‐house egg banks or the potential for eggs to be shipped to clinics both within and across countries, reducing the need for either egg providers or recipients to travel (Coccia et al., 2020). These new forms of frozen egg banking allow clinics and other intermediaries to ‘exploit the *divisibility* of frozen oocytes’ (Waldby, 2019, p. 131), making possible new options on how to deal with donated tissue. New methods of storage and shipping mean that eggs retrieved from one retrieval cycle can potentially increase profit beyond that cycle; a change that may be attractive to clinics in terms of efficiency and profitability (Van de Weil, 2020; Waldby, 2019). Clinics are expanding their egg donation programmes as a result; recruiting more egg providers and potentially becoming increasingly motivated to obtain larger numbers of eggs from each individual (Tober & Pavone, 2019). However, this shift has received little critical attention in the European context. This may be partly due to the long‐standing assumption that in Europe, human gametes are beyond commercialisation. In particular, whilst attention has been given to the use of egg freezing by women who store their own eggs for later use (Baldwin, 2018; Gürtin et al., 2019), little work has considered the perceptions and experiences of this changing landscape amongst women who provide their eggs for use in donation cycles.

There has been a relatively modest focus on providers’ experience on the *process* of egg donation, including egg retrieval and subsequent management practices. What work there is highlights the challenges of informed consent (Thaldar, 2020; Tober et al., 2021), success or failure of eggs in reproductive terms (Randal, 2004; Waldby, 2019) and the intricacies of donor selection practices (Almeling, 2011; Molas & Perler, 2020). A small number of studies have demonstrated how the context of egg donation can impact the experiences of egg providers. For example, one UK‐based study of the identity‐release donation model demonstrates that egg providers would have liked more information about the outcomes than they were given (Graham et al., 2016). Whilst a US‐based study highlights how individuals can have different bodily experiences dependent on whether they are providing eggs for their own IVF or to donate for use in someone else’s treatment (Almeling & Willey, 2017). That experiences appear to be context‐dependent makes comparative research especially valuable. Furthermore, there remains a need for a more developed understanding of women´s experiences in light of the shifts brought about by the expansion of vitrification and the potential new avenues for commercialisation it presents.

This article draws on data from a large‐scale, comparative project designed to explore egg donation in the European context. It aims to contribute to current understandings of women’s experiences of providing eggs for use in third‐party treatment cycles. Our work is informed by Healy´s research on variations in blood donation across Europe (2000). Healy focuses on how different ways of organising donation, rather than differences in individual motivations, shape particular ‘donation regimes’ (Healy, 2000). In this sense, we specifically explore the ways in which socio‐technical regimes shape and are entangled with individual meaning‐making (see also Almeling, 2011; Mohr, 2018; Perler & Schurr, 2021; Wahlberg, 2018) and aim to highlight the ways women perceive their actions and those of clinics within this broader socio‐political framework. We consider how the situatedness of egg donation shapes women’s experiences and accounts, and therefore what becomes ‘thinkable and sayable’ (Prainsack & Wahlberg, 2013, p. 341). In this article, we discuss experiences of egg retrieval in the context of an increasing use of vitrification: first, we focus on the information providers were given about the number of eggs retrieved and the number of recipients for their eggs. In order to explore questions of growing commercialisation in egg donation, we also discuss how a potential increase in profit arising from the use of eggs is seen by egg providers.

## STUDY DESIGN AND METHODS

The study was developed to consider the social, political, economic and moral configuration of egg donation for fertility treatment in the UK, Belgium and Spain and within this, to explore the changing landscape of egg donation. Europe was selected as the site for the study because of the relative lack of research relating to the organisation and practice of egg provision in this context and due to the unique constellation of regulatory oversight for member states that are subject to the requirements of the EU Tissue and Cells Directive, which requires a position of non‐commercialisation. Whilst they operate under the same broad, regulatory directive, our three national cases represent distinct ‘donation regimes’ (Coveney et al., 2022; Healy, 2000), with varying approaches to the organisation, practice, regulation and commercialisation of egg donation.

The project was conducted in four stages. Phase one involved policy mapping (see Hudson et al., 2020), stakeholder interviews and a literature review. Phase two involved the analysis of clinic websites (see Coveney et al., 2022). Phase three involved qualitative interviews with professionals and egg providers to understand perceptions, experiences and moral reasoning amongst those involved, and phase four involved stakeholder workshops and dissemination activities. This article reports on data from phase three interviews with egg providers. We prefer the term egg ‘provider’ to describe the person giving eggs, which does not prejudge the intention of the act. We use both ‘egg provision’ and ‘egg donation’ in this article to describe the practice in more general terms, for ease of expression and to reflect the wording used in the field (see Lafuente‐Funes, 2020, for a critique of the language of ‘donation’). Ethical approval was obtained for the overall project by the host institution (ref #1989) and for the interview studies in each of the countries.

### Case selection

Our three cases share a common position regarding the political legitimation of egg donation as a means of family‐building, but they have each adopted contrasting regulatory models. For example, in the UK, egg providers must be identifiable, whilst in Spain and Belgium they can be anonymous. Known donation can occur in both Belgium and the UK, but not in Spain. Levels of financial compensation also vary across the three countries and have a variable impact in terms of the cost of living. This national diversity makes for a rich source of comparative research, which we outline in further detail below.

#### UK

In the UK, approximately 4500 cycles of IVF with third‐party eggs take place each year, representing around 6.4% of all IVF cycles. There are currently around 1900 new egg provider registrations each year (HFEA, 2021). Egg donation is largely performed within the private sector. Since 2005, UK egg providers must be willing to be identifiable to any offspring born from their eggs at age 18. Treatment can also take place within a ‘known’ arrangement, whereby provider and recipient are known to one another at the outset. Egg sharing is also permitted (whereby a woman undergoing IVF agrees to provide half of her eggs in return for reduced treatment costs). Since 2012, egg providers (who are not egg ‘sharing’) receive £750 compensation per cycle.

Data from the HFEA (Human Fertilisation and Embryology Authority, the UK regulator) shows that currently, egg *freezing* cycles are concentrated in a small number of (mainly London‐based) clinics—in 2016, only three clinics performed over 100 cycles of egg freezing (this includes donor eggs as well as patients freezing eggs for their own later use). Storage periods for frozen eggs have recently been extended from 10 to 55 years in the UK (HFEA, 2022), a change that could dramatically impact this landscape. In the UK, eggs can be used to create a maximum of 10 families, and if donation occurred after 1 August 1991 (the date of the HFE Act), egg providers can be told the number, year of birth and sex of any child. Whilst the UK left the EU on 31 January 2020 (after our data were collected), the regulation of egg donation has remained as it was prior to the UK’s departure; with any EU regulations incorporated into British law via the Withdrawal Bill.

#### Belgium

According to the Belgian Register for Assisted Procreation (Belrap), in 2019, there were 683 cycles of egg donation (Belrap, 2021). In Belgium, whilst egg donation is provided in both public and private hospitals, around 80% of cycles are reimbursed within the Belgian health‐care system. Egg donation is regulated under the Law on Medically Assisted Reproduction and the Disposition of Supernumerary Embryos and Gametes (2007) and can be anonymous (where no identifying information is shared between the egg provider and recipient), known, via egg sharing, or using a practice known as ‘cross‐over’ donation (whereby the recipient brings a donor to the clinic in order to receive an anonymous donation from a different donor). Egg providers in Belgium are compensated for their time and efforts, and compensation varies from clinic to clinic ranging between 500€ and 2000€ (Pennings et al., 2014). It appears that most commonly, a fixed amount of 1000€ is paid. In 2019, 250 cycles of treatment were initiated using previously frozen donor eggs in Belgium: this equates to 36% of egg donation cycles. The storage of frozen eggs is regulated in Belgium by the Law on Medically Assisted Reproduction and the Disposition of Supernumerary Embryos and Gametes (2007) and eggs can be stored for 10 years (however, this relates to a patient’s own eggs and not donor eggs). The maximum number of women who can receive donated gametes from one person is 6, with no limit on the number of children.

#### Spain

Spain is the largest provider of third‐party eggs in Europe; providing 45% of all cycles per year (Wyns et al., 2021). It is also the main destination for Europeans seeking eggs (Culley et al., 2011; Krolokke, 2014). In 2019, 35,674 embryo transfers in Spain involved donated eggs, and over 40% of babies born from IVF carried out in the country come from third‐party eggs (SEF, 2019). There are no data on the number of egg providers, as the donor registry (established in law by 1996) is still not fully functional. The average number of eggs retrieved from providers is increasing, currently 19.6 eggs per cycle (SEF, 2019). The majority of clinics operate within the private sector, which accounts for almost all treatments with third‐party eggs. Whilst some egg donation takes place in public hospitals, this is limited. The 2006 Law on Human Assisted Reproduction does not specify the time eggs and embryos can be kept frozen, neither does it specify the amount of information egg providers can have about the outcome except in relation to identifiability: egg donation in Spain is strictly anonymous. Egg providers receive between 800€ and 1300€ in compensation per cycle. Spanish clinics were early adopters of egg freezing: by 2013, they performed 61.6% of all cycles in Europe (Shenfield et al., 2017). There is a limit on the number of babies born from a single provider (6), but without a registry, it has been difficult to keep track. Even though Spain is subject to the same Directive as other countries in the EU, authors have pointed to the markets and business models that have evolved specifically around egg provision in Spain (Degli Esposti & Pavone, 2019; Lafuente Funes, 2017, 2020; Molas, 2021).

### Data collection

Qualitative, semi‐structured interviews were conducted in all three countries with egg providers who had provided eggs since 2012 (the date of the last major policy change—a national increase in UK compensation—across any of the countries). We included individuals who had volunteered to provide eggs but did not complete a cycle in order to capture the experiences of those who had been rejected or had dropped out. Interviews were conducted between 2018 and 2020. Prospective participants replied to a call distributed through social media, fertility clinics, fertility patient organisations and agencies (via their websites, social media pages, e‐mail, flyers or posters). They were asked to complete an expression of interest which was also used as a means to ensure sample variability. Participants were then contacted by researchers leading the country‐specific fieldwork to arrange the interviews, which took place in a range of venues, including homes, hospitals/clinics, and public venues such as cafes, based on participants’ preferences.

Interviews were conducted in two parts. First, using a semi‐structured interview guide, egg providers were asked about their routes into egg donation, their experiences with clinics and agencies, their views on the use of their eggs and their knowledge and feelings about the outcome. In the second part of the interview, egg providers were presented with a number of scenarios relating to the *possible* use of eggs within fertility clinics, including questions about storage, movement and potential commercial practices. Interviews lasted between 1 and 2.5 h and were recorded and transcribed verbatim in the local language (English, Spanish, and Flemish). Data presented in this article are drawn from both parts of the interviews.

### Sample

Seventy‐five interviews were conducted with egg providers: 29 in the UK, 21 in Belgium and 25 in Spain. Data collection tools and summaries are available at the UK Data Service (SN #855467). Table 1 provides an overview of their age, education, employment status and number of donation cycles, organised by country. Table 2 provides an overview of the different types of donation possible in each country and represented in the sample.

**TABLE 1:** Participants by country, age, education, employment and number of cycles

		UK (*n* = 29)	Belgium (*n* = 21)	Spain (*n* = 25)
Age at interview (years)	Range	22–42	26–42	18–36
	Mean	32.5	32.6	27.1
Education level (*n*)	Secondary school	11 (38%)	8 (38%)	9 (36%)
	Graduate/postgraduate	18 (62%)	13 (62%)	15 (60%)
	Missing data	‐	‐	1 (4%)
Employment status (*n*)	Full time	16 (55%)	17 (80%)	12 (48%)
	Part time	8 (28%)	3 (15%)	4 (16%)
	Not in employment	1 (3%)	1 (5%)	8 (32%)
	Missing data	4 (14%)	‐	1 (4%)
Cycles donation (*n*)^a^	Range	1–6	1–3^b^	1–8
	Mean	1.62	1.5^b^	2.2

^a^ Only those who had completed a cycle.

^b^ Based on *n* = 20 women: excludes one Belgian provider who recollected donating between 15 and 20 times and is an outlier.

**TABLE 2:** Types of donation: permitted and represented in the sample

Type of donation	UK (*n* = 29)	Belgium (*n* = 21)	Spain (*n* = 25)
Anonymous	n/a	14 (67%)	22 (88%)
ID‐release	12 (42%)	n/a	n/a
Known	3 (10%)	1 (5%)	n/a
Cross‐over donation	n/a	1 (5%)	n/a
Egg‐share	5 (17%)	‐	n/a
Did not donate	4 (14%)	3 (14%)	3 (12%)
Multiple forms of donation	5 (17%)	2 (9%)	n/a

### Data analysis

Qualitative thematic data analysis was conducted in four stages, supported by a qualitative software package. First, interview transcripts were subject to a process of open coding and memo‐writing (Braun & Clarke, 2006), carried out in the local language. Next, a common catalogue of codes was developed in English, based on this initial analysis. A series of analysis meetings were conducted with the whole project team across three countries in English, in order to generate and agree on this catalogue. The catalogue provided a common, overarching coding structure for use across the cases whilst allowing attention to be paid to the particularities of each context (Braun & Clarke, 2021). The data were analysed per country using this coding catalogue. Finally, country‐specific analysis reports were generated by drawing on themes pertinent to the topic (in this case, egg retrieval and usage). For this article, themes relating to women’s accounts of the numbers of eggs retrieved, the number of recipient cycles they might be used in and their views about a number of ways profit could potentially be made from eggs were subject to stage four analysis. Two core themes: ‘Keeping count? What egg providers knew about the number and use of eggs retrieved’ and ‘Who benefits? Concerns about over‐commercialisation in clinics’ use of eggs' were generated in dialogue with the literature and are reported below.

## FINDINGS

### Keeping count? What egg providers knew about the number and use of eggs retrieved

We asked women about their recollection of and knowledge about the number of eggs retrieved during their cycles and what they knew about how the eggs were used. This included the number of recipients or cycles their eggs were used for. Women described what they had been told by doctors and nurses or by other clinic or agency staff. Women’s experiences varied across the three countries, and what they knew and how they felt about it depended on both national policy around information‐giving for egg providers as well as local and/or clinic practice. In the UK, women reported being informed about the cycle and had the most direct access to information, with clinic staff volunteering information or responding to requests. In Belgium, egg providers commonly knew about the number of eggs retrieved but less about the number of recipients or cycles; however, Belgian egg providers described an expectation that their eggs would be given to more than one recipient. In Spain, women were least likely to know about the number and use of their eggs as clinic staff tended not to provide information and some egg providers felt reluctant to ask, though this varied by clinic. We expand on data from each country below.

### UK—Information sharing as the norm

In the UK, the majority of egg providers described being told the number of eggs retrieved on the day, either by nurses or doctors volunteering the information or upon request. This is in keeping with UK policy, which permits egg providers to know the number of children, their biological sex and the year of their birth. Annabelle (ID‐release, age 29, 6 cycles), who provided eggs at different clinics, reported that in one clinic ‘they told me as soon as I was awake how many they had got’, and in another ‘when I asked’. Madeleine (ID‐release, age 29, 1 cycle) also recollected that she did not ask or need to ask for the number of eggs retrieved but that the clinic staff openly talked about it in her presence, thus making the information available:

You [could] just hear “oh we got 13 eggs”

Madeleine (ID‐release, age 29, 1 cycle)

In the majority of cases, UK egg providers had the option to learn additional details about the outcome of the donation. For instance, Susan (ID‐release, age 31, 4 cycles) was given a choice about to what extent she would like to be informed about the outcome. ‘When you initially start, they ask you if you would like to know, so you can say “no, I am not interested, just have it and I’m happy if everything is alright”’. However, Susan recalled that later she did want to know and was therefore happy to take up the clinic’s offer of additional information.

The specific level of detail given varied across the UK sample; some were kept informed about the number of eggs maturing during stimulation, how many were retrieved and how many of those were considered to be of good (enough) quality. Emma (egg‐share, age 31, 1 cycle) recalled how the nurse showed her how things were progressing on the ultrasound:

“there was like 16 follicles”, and on the retrieval day, “nine came out”.

(Emma)

UK egg providers also commonly knew the number of recipients for their eggs (in cases of identity‐release donation—in known donation, it was usually one specific recipient):

I knew I was donating to two couples.

(Gaby, ID‐release, age 33, 3 cycles)

The clinic… said that anything over five [eggs] would be split between two recipients.

(Annabelle, ID‐release, age 29, 6 cycles)

In cases where cycles did not go according to plan, women in the UK were commonly informed about changes, such as a reduction in the number of recipients:

[I had two recipients], but then I didn’t find out until after everything that I only ended up donating to one. Apparently one of the ladies dropped out. I don’t know which one but, yes, so one lady got all 13 eggs.

(Madeleine, ID‐release, age 29, 1 cycle)

Whilst information sharing with UK egg providers was the norm, clinics varied to some degree in whether they shared the information routinely, or if individuals needed to ask for it.

### Belgium—Trusting clinics in the absence of detailed information

In Belgium, most egg providers recalled being informed about the number of eggs that were retrieved but not about other aspects (i.e., numbers of recipients). As in the UK, there was some variation in how they received this information with some needing to ask and others being told straight away:

I am well aware how many [eggs they retrieved], the first time, ehm, it was 11, and the second time it was 13.

(Louise, 1 cross‐over donation, 1 anonymous donation, age 31, 2 cycles)

As in the UK, some Belgian egg providers were interested and knew additional details about how many eggs were retrieved, selected and finally fertilised:

I believe they retrieved 25 and 17 were useable. That means, 17 eggs were good. (…) They then tried to fertilize 17, and I think, I think 11 fertilised.

(Mona, known donation, age 30, 1 cycle)

Belgian egg providers said they knew little about how many recipients received their eggs (the majority were donating as part of an anonymous cycle) or how many cycles were performed. Instead, information was described as being given in general terms, rather than being specifically about what happened to *their* eggs. For example, several women indicated they were told the eggs would be divided into batches of six: one recipient would receive six, and any supernumerary eggs would go to other women. The commonly held view was that the eggs were meant to help ‘other *women’* (plural) and trusted clinics to enact this:

That [eggs going to several women] is what I have in my mind, that it happened that way.

(Reneé, anonymous donation, age 32, 1 cycle)

However, this was not always clear to them:

I do not know this very well actually. It seems to me that on the one hand, it is weird if my eggs, “my eggs” [laughs], my eggs would be divided over…. Yes, now there are 13… Suggest over 13 women. That gives a bit of a weird image. But yes… On the other hand probably, they will not… for one woman… Yes, I do not know actually. I do not know how that is done exactly. I thought per woman 6 eggs… But I do not know.

(Lina, anonymous donation, age 33, 1 cycle)

Only two women said that they were informed up front that at least two women would receive their eggs:

So the fresh ones, always six fresh ones, and then six frozen. And the leftovers also, one who gets all the leftovers or so? [laughs] That is all I know.

(Ines, anonymous donation, age 34, 3 cycles)

Four women described how they had assumed there would only be one recipient. Some women were not given any precise information about the number of recipients or cycles, but did not mind this, such as Chloé (anonymous donation, age 33, 1 cycle), who was nevertheless hoping that her donation would be shared amongst several recipients. ‘They only said “we got 15 (…) I don't know about [the number of recipients]. No, they also said ‘our story ends now”’. Chloé agreed that this would be the limit of information provided to her and added:

I hope they can make several people happy with that.

(Chloé)

Most egg providers in Belgium were informed about the number of eggs they provided but had little precise information about the number of recipients or cycles that their eggs were used for. However, in Belgium, women tended to assume, or were told in the abstract, that their eggs were given to more than one recipient.

### Spain—‘Closing doors’: Limited information and differing levels of trust

In Spain, participants described variation between clinics in relation to information‐giving. Most women did not recall being given any information about the number of eggs retrieved, though six described being directly informed. Some providers wanted to know but were not told, and others asked directly how many eggs were retrieved but did not receive a clear answer:

They said “quite enough”, but that is all

(María, anonymous donation, age 18, 1 cycle)

Others did not ask because they felt they were not entitled to know, whilst a few mistrusted the information from clinics:

They tell you that they will take around 5 or 6, something like that. But then you look the ultrasound and you can see you have many more… I think they never tell you the truth about this.

(Valeria, anonymous donation, age 23, 4 cycles)

One egg provider suggested that clinics could be reluctant to mention the number out of fear that they would ask for higher compensation:

Because maybe if they tell me “Look, we retrieved, I do not know, 14” I would be like “Fuck! If you have 14, if we count it by the egg… and you only give me 1000 euros then maybe…”

(Luna, anonymous donation, age 29, 1 cycle)

Nonetheless, some clinics that did not give information did not hide it either, and as a result, some providers overheard the number discussed in professionals’ conversations. Two women obtained this information when clinic staff told them there were not ‘enough’ eggs and encouraged them to donate again. Mia was told they:

only retrieved five and they need at least ten.

(Anonymous donation, 28, 1 cycle)

More generally, Spanish egg providers described expecting fewer eggs to be retrieved than the national average (19 eggs per cycle), and many had unclear ideas of what might be considered a ‘normal’ amount.

In general, Spanish egg providers had little to no information about what happened to the eggs after retrieval. In some cases, mandatory anonymity was seen as the reason for this lack of information. For example, Bruna (anonymous donation, age 32, 5 cycles) wanted to know if a pregnancy occurred but reported that ‘they say nothing’ and reflected how ‘privacy there is tremendous’. In other cases, this lack of information was seen as protection to ensure that providers did not feel attached to the result. Indeed, Elisa (anonymous donation, age 23, 1 cycle) reflected how she tended to ‘overthink’ and not knowing helped her ‘stop [overthinking], for my mental health’. There were some exceptions to this lack of information; for example, one participant said she could have asked if a pregnancy was achieved, but preferred not to:

They say: The only thing we can tell you… as she [the recipient] cannot know anything about you, nothing, nothing… the only thing we can tell you from her side is if she could conceive.

(Victoria, anonymous donation, age 19, 1 cycle)

Others were indirectly told their eggs worked, as in Lucía’s case; she was told this was the reason they called her to donate again:

They tell you: “are we going to do the whole hormone treatment if they are of no use?” if they call you to do it again is because everything went well. And for me, for now, in every clinic I’ve been at, all went well.

(Lucía, anonymous donation, age 29, 4 cycles)

Some providers thought that the eggs went to one recipient, whilst others had the idea they could be distributed among more recipients. However, in general, they had little information. Some providers were content with this, although many would have liked to know a bit more:

Well there is always the wish to know… not the persons, but how many couples have I helped.

(Alejandra, anonymous donation, age 30, 2 cycles)

Having discussed women’s experiences and what they were told by staff in relation to the use of their eggs, we also asked them to consider a number of possibilities regarding the ‘in principle’ use of eggs once they were donated. We were interested to know women’s views about the management of eggs in the context of vitrification. We gathered data on providers’ views about potential uses of the eggs by clinics, such as dividing eggs among multiple recipients or transferring them to other clinics (nationally or internationally) for an additional fee (so‐called ‘transnational egg donation’). Thus, following existing work on the commercialisation of eggs (Waldby, 2019), the second theme from our data presents the women’s reflections on the potential for clinics to make profit from eggs, specifically beyond the initial retrieval cycle.

### Who benefits? Concerns about over‐commercialisation in the use of eggs by clinics

Whilst the majority of participants in all three countries were opposed to the generation of profit from eggs, there were different reasons for this. In the UK, egg providers were more likely to emphasise that egg donation was not about making money, but about the act of *donation* and should not therefore be commercialised. Some women did express that some kind of profit‐making was inevitable. In Belgium, the most common narrative was that whilst egg donation is not about making money, the recouping or ‘reimbursement’ of costs associated with the extraction and treatments were seen as legitimate. In Spain, most providers took profit‐making practices as a given but were resistant to the idea that clinics could or should be enlarging their profit margins, or making ‘extra’ profit.

#### UK—Giving eggs as ‘donation’: Concerns about over‐commercialisation

The majority of UK egg providers opposed the idea that egg donation should be or could become about making profit. Women’s opposition was firmly grounded in the perception that egg *donation* is not or should not be about making profit. Some UK egg providers felt that ‘the clinics get paid more than enough anyway’ (Esther, egg‐share, age 33, 1 cycle), whereas others pointed out that eggs ‘[are] not something to be sold, they’re something to be donated’ (Rose, did not donate, age 28) and raised concern that it would be contradictory or hypocritical if clinics were permitted to sell eggs (to another clinic), while egg providers could only *donate* on an altruistic basis with compensation for their time and efforts:

…you can't even sell your own [eggs] because that's compensation for your expenses and stuff.

(Carol, ID‐release, age 25, 1 cycle)

The clinic should not do that, maybe personally because I don't want them making money off of my eggs. No, because if I have gone through all of that, no, I don't think you [the clinic/a third person] should be making money, personally.

(Emma, egg‐share, age 31, 1 cycle)

Some UK egg providers were neither in favour nor opposed to clinics making extra profit:

We’d like to think that this [egg donation] wasn’t a monetised thing. It is and I am paying for my treatment and I understand that things cost money. I suppose you could say that eggs are quite a valuable resource so I wouldn’t necessarily be against the payment side of it. It would be lovely if people just gave eggs and didn’t expect anything in return, you know, the clinics, but I understand that they run a business.

(Kylie, did not donate, age 32)

Finally, some women reflected on how the possibility of making more money out of each donation (e.g., by batching eggs for use by multiple recipients) could serve as an incentive to retrieve more eggs, which in turn could put egg providers at risk of ovarian hyperstimulation:

They were just desperate to get as many eggs out of me as they could. It’s possible that this transfer of eggs [to another clinic] if they had sent those surplus eggs elsewhere it might encourage them to overdo hormones a bit to get 27 eggs (…) there is a bit more potential for abusing that.

(Gaby, ID‐release, age 33, 3 cycles)

#### Belgium—A pragmatic approach: The recouping of clinic costs

In Belgium, women had mixed views about whether clinics should be permitted to generate income from eggs. Two thirds of women were not in favour of a clinic receiving additional funds from the transfer or ‘sale’ of their eggs to another clinic:

No, I am bothered by that. (…) Just… I have trouble with financial compensations. That is very sensitive to me. Financial compensations, I always immediately think about euh, shopping (laughs).

(Lucie, cross‐over donation, anonymous donation, known donation, age 42, 3 cycles)

Extra compensation for the donor would be ok, but not for the clinic.

(Ines, anonymous donation, age 34, 3 cycles)

However, a proportion—around a third—of the Belgian participants were OK with the idea of the clinic recovering the costs of transferring eggs to another clinic. Their reasoning was that the fee for the eggs was seen as covering expenses, in form of capital and time, incurred by the clinic to retrieve the eggs:

Yes, they have made expenses.

(Camille, anonymous donation, age 27, 1 cycle)

Yes. Because they also put their money and time in it.

(Eva, anonymous donation, age 32, 2 cycles)

This was presented as a condition whereby the extra income should not exceed the costs incurred by the clinic in retrieving the eggs (i.e., they should not ‘profit’ from the eggs). Axelle considered additional payment from one clinic to another for a transfer of eggs acceptable, since: ‘they should share in the costs’ (anonymous donation, age 32, 6 cycles).

As in the UK, the majority of egg providers in Belgium were not in favour of ‘profit’ being derived from eggs but considered an additional payment to the clinic a potentially legitimate way for clinics to recoup their costs.

#### Spain—Profit‐making: Generalised assumptions of a commercial approach

As in the other two countries, most egg providers in Spain disliked the idea of clinics making too much profit from their eggs. Some providers were uncomfortable with clinics sending eggs to other clinics because they thought they were donating to either a single recipient or a single clinic, trusting the clinic to enact this:

I donate my eggs to give them to one patient in this particular clinic. If they have surplus and then they sell them… because that’s what I would think they are doing, sell them in case there is economic profit for something you did for a *specific patient* in their clinic: in particular (Ana’s emphasis).

(Ana, anonymous donation, age 24, 3 cycles)

They [the clinic] are gaining more money… I mean, they already make profit with the first process… (…) I would see it like a weird egg trafficking thing… very weird.

(Victoria, anonymous donation, age 19, 1 cycle)

In that sense, some Spanish egg providers were opposed to what they saw as clinics profiting twice from their eggs, which was seen as an illegitimate activity. Most felt that the presence of commercial logics in fertility treatment were a given but nevertheless problematised this as over‐commodification:

I do not think that I am earning money for the eggs. Therefore, I would think it is wrong that the clinic gets paid for the eggs.

(Constanza, anonymous donation, age 18, 2 cycles)

Only two participants felt this was acceptable practice, and as with the Belgian participants, this was linked to costs seen as associated with the process. Excessive profit‐making was seen as unjust, both towards the women providing the eggs and fertility patients paying for treatment:

When a person goes to a reproductive clinic, because she wants to be a mother, she is already making an initial payment. If she is already paying and you search a donor for her, she already paid you. Why do you need to get money again if you already charged that service? No. That is not fair. And I mean it because those are *my* eggs. And those are the eggs that this woman [the recipient] has *bought*, even if it sounds ugly to say it out loud (Alejandra’s emphasis).

(Alejandra, anonymous donation, age 30, 2 cycles)

Some Spanish egg providers who had negative experiences felt that overcommercialisation could be considered abusive towards egg providers. In this vein, Martina (anonymous donation, 18, 1 cycle), whose treatment resulted in ovarian hyperstimulation, suggested that the clinic treated her as if she were: ‘the goose that laid the golden eggs’.

## DISCUSSION

Little work to date has considered egg providers’ views about the intricacies of the practice of egg donation and the processes involved in the extraction, management and use of eggs. There has been even less focus on the European context, where common regulation theoretically inhibits the commercialisation of human tissue, including egg cells. Simultaneously, the introduction of vitrification, along with growing use of third‐party eggs by fertility clinics globally, are impacting practices and reshaping the egg donation landscape. Vitrification complicates egg donation practices in a number of ways. It opens up new avenues for the use, storage and movement of eggs previously unseen, and along with this, the potential for increased profit‐making, renewing discussion over bodily commodification (Waldby, 2019). The ability to batch frozen eggs and to divide them amongst recipients disrupts existing imaginaries of egg donation, common in many (but not all) contexts, being a bodily ‘gift’ given from one woman to another (Coveney et al., 2022; Gezinski et al., 2012; Shaw, 2008). As a result, changing practices also raise new questions about how much information providers should be or are given about the potential use of their eggs once they provide them to a clinic, and about whether they should be involved in decisions regarding what clinics can do with their eggs in the future.

Comparing experiences in the context of different national donation ‘regimes’ allows an exploration of the ways in which varying national practices and policy landscapes shape what information is given to egg providers and, in turn, how women experience and make meaning of the process. As our data demonstrate, the ways in which information is given in each context are important in shaping what is ‘thinkable and sayable’ (Prainsack & Wahlberg, 2013, p. 341), and therefore for how donation itself is shaped. This moves us away from a position of thinking about ‘donation’ as a unitary or homogenous entity, or one whose form depends on individual ‘motivations’ and instead gives rise to an understanding of egg provision as a specific, embedded and heterogenous practice. It also permits attention to be given to the voices of egg providers, who are often omitted—as biocitizens (Happe et al., 2018)—from debates about the politics and policy surrounding egg donation (Haimes, 2015; Heidt‐Forsythe, 2018). In the context of differing donation regimes, we can consider how providers’ experiences shift in relation to the new range of options opened up by vitrification, whether individuals are informed about these options and what this tells us about the wider regulatory regime prioritised in each country.

UK egg providers appeared to be the most well informed about the number and use of their eggs; a finding that is potentially explained by the UK regulation, which allows them to be told the number of offspring born from their eggs. The UK context—where egg providers will eventually be identifiable—potentially gives rise to a situation in which information‐giving is the norm. It follows that clinic staff are able and willing to offer women information about the retrieval process and number of recipients. In general, the UK cultural and regulatory model of ‘openness’ has generated a context in which women tended to have more information and felt entitled to it (Gilman & Nordqvist, 2018; Graham et al., 2016).

In Belgium, most egg providers were informed about the number of eggs they provided but had little precise information about the number of recipients. Information‐giving appeared to stop with the process of retrieval, and the intended use of the eggs was not shared. The majority of Belgian egg providers in our sample were providing their eggs in an anonymous cycle, and this may have shaped their experience. This finding aligns with the framing of egg donation in Belgium as a form of biomedicalised tissue donation (Coveney et al., 2022), in which the donation is framed and discussed as akin to other forms of bodily donation. It also aligns with the idea of the ‘clinic’ at times needing to be held separate from the ‘marketplace’, given that most donation takes places in the publicly funded health‐care system (Haimes & Williams, 2018). Despite the lack of specific information, Belgian women tended to assume, or were told in the abstract, that their eggs would be given to more than one recipient, and in general appeared to be content with this level of information.

In Spain, many participants described having been given less information about the cycle than they wished; a finding also confirmed by Molas and Perler in their ethnographic work in Spain (2020). Most participants either knew nothing or had unclear ideas about the average number of eggs to be extracted, not to mention how many were actually retrieved. Variability in what women were told in Spain may be related to the existence of flexibility within Spanish regulation, which allows for differing practices. However, ultimately, it appeared that less information was given in Spain than in the UK and Belgium. Whilst women in Belgium similarly received little direct information about the use of their eggs, some Spanish egg providers expressed unease about this and were more likely to express suspicion about the motivations of clinics than women in the UK and Belgium.

Talking about the income generation and profit‐making practices of clinics with egg providers provides a novel lens through which to analyse ideas about commercialisation; showing how the different ways egg donation is organised in each country shape how commercial questions may be understood, critically addressed or taken for granted. In the UK, egg provision was embedded within a cultural framing of altruistic donation, which co‐existed with an assumption amongst some women that profit was being made; a finding that can be related to the economic reality that the majority of egg donation takes place within the private sector (Haimes & Williams, 2018). In Belgium, where clinics largely reside in the public sector, questions of clinic practices and profit‐making were accompanied by a feeling of trust towards clinics. Here, the main argument was that egg donation should not be (and is not) about making *profit* (for anyone involved) but might be at least partially linked to the economic model of ‘reimbursement’ for costs incurred—which tended not to be seen as a payment. In contrast, in Spain, commercial logics were taken as given. Spain is the main European market due to the availability of eggs and the majority of cycles occur in the private sector. Here, participants showed different levels of discomfort with the idea of clinics ‘making money’ from eggs—and specifically with them expanding profit via batching practices. Within this view, some providers saw egg donation as a commercialised practice in which profit was unevenly distributed. These varied cases illuminate how, within the organising category of ‘egg donation’, we find differing donation regimes (Coveney et al., 2022; Healy, 2000), which produce varying narratives, framings and experiences. In the UK, we might think about this framing as a system of ‘informed gift‐giving’, in Belgium, as a form of ‘trusted tissue exchange’ and in Spain, as a system of ‘closed‐door, market‐driven donation’.

These findings demonstrate the embedded and contextualised nature of biomedical practices and their possible commercialisation. Whilst there was an acceptance that the provision of eggs for use in fertility treatment was part of a wider set of practices and interests, which often rested on the generation of profit, on the whole, egg providers (particularly in the UK and Belgium) were keen to keep their eggs ‘beyond trade’ (Hoeyer, 2009). This was multi‐layered: at times related to the value attributed to the act of ‘donation,’ but also appeared when some women talked about the potential double standard that they cannot ‘sell’ eggs, and therefore neither should clinics. Whilst most egg providers saw a trade in eggs as undesirable, the reasons for this were nuanced and context‐dependent. Similarly, the degree to which egg providers maintained an interest in what happens to ‘their’ eggs— that is, to enact a particular form of biocitizenship—varied and was shaped by local regulation (Haimes & Williams, 2018). Our findings suggest the need to reconceptualise ideas about the commodification of body parts and the existence of reproductive bioeconomies in order to highlight nuances, multiplicity and contextual variation.

## CONCLUSION

Our data demonstrate the need to engage in a more thorough and critical analysis of the experiences of egg providers in the context of a rapidly transforming global, reproductive bioeconomy. It extends our understanding of the varied ways in which egg donation is managed at the national level and the ways in which donation regimes work to produce specific experiences, expectations and subjectivities. It shows that egg donation itself varies internationally, with the production of a range of regulatory and commercial realities evident. Our findings about women’s perceptions about profit‐making practices in Europe raise questions about how and whether egg donation practices may be increasingly out of step with existing policy, as well as with the views of key stakeholders (those providing eggs), especially in Europe where the commercialisation of human tissue is forbidden. Our findings raise important questions about information‐giving and, by extension, processes of informed consent. In a context in which donated eggs can increasingly have multiple destinies due to both technological and regulatory changes, it may be timely to consider not only ways in which to inform egg providers but also to consider if they should be permitted to make decisions about the tissue they provide.

## AUTHOR CONTRIBUTIONS

**Sara Lafuente‐Funes**: investigation, methodology, analysis, writing—original draft preparation. **Christina Weis:** investigation, analysis, writing—original draft presentation. **Nicky Hudson**: funding acquisition (lead), conceptualisation, methodology, project administration and supervision (lead), writing—review and editing. **Veerle Provoost**: funding acquisition, conceptualisation, methodology, project administration and supervision, writing—review and editing.

## Data Availability

Data collection tools and summaries are available at the UK Data Service (SN 855467).
